# Uncoupling flagellum formation and maintenance

**DOI:** 10.1186/2046-2530-4-S1-O17

**Published:** 2015-07-13

**Authors:** C Fort, J Santi-Rocca, P Bastin

**Affiliations:** 1 Trypanosome Cell Biology Unit, Institut Pasteur, Paris, France

## Objective

The role of Intraflagellar transport (IFT) in the construction of cilia and flagella is well established in numerous species. In contrast, its involvement in flagellum maintenance has only been shown in *Chlamydomonas*. Here, the protist *Trypanosoma brucei *was used as a model to investigate the role of IFT in flagellum maintenance. This organism has the advantage to maintain the existing flagellum whilst assembling the new one during the cell cycle. Knocking down the expression of any IFT protein inhibits flagellum formation in trypanosomes. Nevertheless, 25% of the cells still possess a flagellum of variable length. It was initially proposed that this was a consequence of RNAi that targets mRNA and not protein, hence IFT would still be active in these flagella.

## Methods

The *IFT88^RNAi ^*and *IFT140^RNAi ^*strains were transfected with a construct allowing endogenous tagging of IFT81, another IFT protein, with YFP. IFT was monitored at different stages of RNAi and cells were also analysed by electron microscopy.

## Results

New flagella of intermediate length are still assembled at early time points of RNAi but IFT trains are less frequent and appear smaller in the *IFT88^RNAi ^*strain. Later on, the new flagellum is not formed but the old flagellum, assembled before RNAi was triggered, remains in place. Nevertheless, IFT is not detected anymore, in agreement with the dramatic reduction in the number of IFT trains observed by electron microscopy. In the *IFT140^RNAi ^*strain, an excessive amount of IFT material is detected in their old flagellum and photobleaching experiments revealed that this material does not traffic. Therefore IFT is either missing or arrested in IFT-B and IFT-A mutants respectively.

## Conclusion

In both cases, absence of active IFT is not accompanied by flagellum shortening meaning that IFT is not necessary for flagellum length maintenance. Proteomic comparison of flagella is in progress and suggests modification of the flagellum content in the absence of active IFT.

